# Spectral and structural comparison between bright and dim green fluorescent proteins in Amphioxus

**DOI:** 10.1038/srep05469

**Published:** 2014-06-27

**Authors:** Erin K. Bomati, Joy E. Haley, Joseph P. Noel, Dimitri D. Deheyn

**Affiliations:** 1 Marine Biology Research Division, Scripps Institution of Oceanography, University of California, San Diego, La Jolla, CA 92037 USA; 2 Materials and Manufacturing Directorate, Air Force Research Laboratory, Wright Patterson Air Force Base, OH 45433; 3 Howard Hughes Medical Institute, Salk Institute for Biological Studies, Jack H. Skirball Center for Chemical Biology and Proteomics, La Jolla, CA 92037

## Abstract

The cephalochordate Amphioxus naturally co-expresses fluorescent proteins (FPs) with different brightness, which thus offers the rare opportunity to identify FP molecular feature/s that are associated with greater/lower intensity of fluorescence. Here, we describe the spectral and structural characteristics of green FP (bfloGFPa1) with perfect (100%) quantum efficiency yielding to unprecedentedly-high brightness, and compare them to those of co-expressed bfloGFPc1 showing extremely-dim brightness due to low (0.1%) quantum efficiency. This direct comparison of structure-function relationship indicated that in the bright bfloGFPa1, a Tyrosine (Tyr159) promotes a ring flipping of a Tryptophan (Trp157) that in turn allows a cis-trans transformation of a Proline (Pro55). Consequently, the FP chromophore is pushed up, which comes with a slight tilt and increased stability. FPs are continuously engineered for improved biochemical and/or photonic properties, and this study provides new insight to the challenge of establishing a clear mechanistic understanding between chromophore structural environment and brightness level.

First discovered in 1961 by Shimomura and colleagues in the cnidarian jellyfish *Aequorea victorea*^1^, the green fluorescent protein (GFP) and its variants have become the cornerstone of fluorescent protein technologies, exponentially expanding the application of fluorescence spectroscopy and imaging in molecular, cellular and developmental biology, as well as the applied fields of biotechnology and bioengineering^2^,^3^.

Critical steps in the wide-spread use of GFP included the elucidation of the three dimensional structure of the wild-type GFP^4^ and the S65T-GFP mutant that resulted in increased fluorescence, photostability and a red shifting the major excitation peak to 488 nm with the peak emission kept at 509 nm^5^. These initial atomic resolution views of GFP highlighted the unusual architecture and key functional groups of this protein family described as ‘paint in a can'^5^. GFP folds into an 11-stranded β-barrel with a single helical segment threaded through the center of the barrel, and capped by several loops at either end of the barrel. A sharp turn places strain on three residues within the barrel-encapsulated stretch, which consequently drives cyclization via a dehydration/oxidation mechanism that requires only molecular oxygen^6^,^7^,^8^. The cyclized chromophore remains covalently attached to the polypeptide chain, and therefore, every cell expressing the *GFP* gene acquires fluorescence. This genetically encoded autonomy and self-assembly makes the *GFP* gene a powerful biological tag for the *in vivo* visualization of a wide array of cellular structures and processes^2^,^9^,^10^.

Since the publication of the landmark crystal structures from the Remington and Phillips groups, respectively^4^,^5^, more than 250 structures of engineered GFP variants from *Aequorea victorea* have been deposited into the Protein Data Bank (PDB), and are described in many publications (www.rcsb.org)^11^. This extensive catalog of three-dimensional structures coupled with biochemical characterization has enabled both structure-guided engineering of the wild-type *Av*GFP and the ability to decipher the effect of random mutations and directed amino acid substitutions on the proteins' optical characteristics^11^. These engineering efforts have produced GFPs with widely varying solubilities, oligomerization states, pH optima, halide and temperature sensitivities, and a diversity of fluorescence intensity, brightness, absorption and emission spectra (e.g.^12^,^13^,^14^). While these engineering efforts produced impressive improvements in a broad array of biophysical parameters, they were mostly associated with production of green fluorescence, while the production of GFP-like proteins fluorescing in the orange-red range of the visible spectrum remaining more challenging^15^.

Some research efforts were also developed towards finding fluorescent proteins from other organisms than the *Aequorea* jellyfish, in order to identify whether Nature could have “engineered” GFPs with other structural backbones, or fluorescent proteins with different structure/s, and hopefully with different (and attractive) biophysical and photonic properties. As a result, an increasing number of GFP-like crystal structures from many different species of cnidarians initially, but more recently also from crustaceans, have been deposited in the PBD. In particular, a red fluorescing GFP-like protein, DsRED, was identified in the cnidarian stony coral *Discosoma*^16^,^17^,^18^, and engineered to yield a set of proteins called mFruits, with colors ranging from honeydew to cherry hues^19^. Beyond their importance for expanding the color spectrum of genetically encoded imaging agents, this more phylogenetic approach revealed fascinating FP properties such as kindling (transition from non-fluorescent to fluorescent protein upon intense green light irradiation)^20^, photo-conversion (changing both excitation and emission spectra upon irradiation with high energy light)^21^,^22^,^23^, and photo-induced protein cleavage^24^. Furthermore, while the β-can architecture is conserved for all GFPs examined to date, novel chromophore structures have been discovered in GFP-like proteins from other cnidarians species^25^. It must be noted, however, that in most species only a few (and mostly one) distinct gene of GFP protein can be found per organism^26^,^27^, thus limiting greatly the comparison of optical and biochemical performances from GFPs naturally co-expressed within the same organism.

Quantum yield (QY), also referred to as the quantum efficiency (QE), is the probability that an excitation of the electronic dipole of the chromophore leads to the emission of a photon instead of a heat dissipating transition as relaxation back to the ground state occurs^28^. QE is a key-factor affecting the GFP “Brightness” defined as the product of the QE and the chromophore's molar extinction coefficient, the latter representing the extent to which a chemical species absorbs light at a given wavelength. Despite extensive engineering efforts, QE improvements remain difficult for most GFP-like proteins. QEs associated with commercially available engineered-GFPs (possessing emission spectra maximums between 500–510 nm) range from 0.53 (TurboGFP) to 0.91 (mM * cm)^−1^ (ZsGreen1)^3^. However, all commercial GFPs exhibit low molar extinction coefficients, resulting in only “modest GFP Brightness” [all expressed in (mM * cm)^−1^]: 37 for TurboGFP, 40 for ZsGreen1, as low as 26 for T-Sapphire, and as high as 41 for Azami Green^3^, thus improving by only a maximum of 121% the brightness of eGFP^9^.

The need for GFPs with improved brightness becomes apparent as researchers push the limits of signal detection. GFPs are widely used to address protein-protein interactions based on single molecule fluorescence detection, or fluorescence shifts from Förster Resonance Energy Transfer (FRET)^29^,^30^,^31^. Using these techniques however is associated with a typical 40% loss of input light (photons), as is the case when using eGFP, which can clearly become a limiting factor for optimal signal detection. Despite the wealth of three-dimensional information available, the specific structural factors modulating GFP QY remain a speculative area of biophotonic research. Ideally, comparing bright and dim GFPs with the same backbone would lead to constructive insights on how to direct engineering for spectral optimization of fluorescent proteins.

We recently identified a family of GFP proteins (bfloGFP) in the cephalochordate, *Branchiostoma floridae*^32^, an invertebrate phylogenetically closest to vertebrates^33^. In this particular species, an animal commonly called amphioxus or lancelet, the 16-member family of fluorescent proteins represents the largest set of GFPs yet discovered in a single species. These GFPs group into six clades, each clade possessing distinct fluorescence intensities, extinction coefficients, and absorption profiles, although always emitting light in the green color when collected from the field^34^, and despite some red fluorescence reported in lancelets by other groups^35^,^36^. Accessibility to such widely varying GFPs from a single organism represents a unique opportunity to investigate natural variation within a single species and the evolutionary consequences for properties of the encoded FPs. In a recent study, we described the spectroscopic properties associated with a representative member of each of four FP clades, showing that the fluorescence intensity is particularly high for some bfloGFPs, while particularly low for others^34^. In the present study, we focus our efforts on the structure-function analysis of a brightly fluorescent member, bfloGFPa1, and a weakly fluorescent member, bfloGFPc1. We present biochemical and spectral characteristics as well as three-dimensional structures derived from protein x-ray crystallography of both proteins, and discuss the structural differences in light of the chromophore environments and the resulting photonic properties of each GFP.

## Results

### Protein purification and crystallization

Brightly fluorescent bfloGFPa1, weakly fluorescent bfloGFPc1, and eGFP were expressed in *E. coli* with thrombin cleavable N-terminal hexahistidine tags, and purified via Ni^2+^-NTA affinity and size exclusion chromatography (SEC). SEC confirmed the monomeric character of eGFP while both bfloGFPa1 and bfloGFPc1 behaved as dimers (Fig. S1). The *Amphioxus* GFPs' peak spectral characteristics were (peak ± FWHM) bfloGFPa1 = 497 ± 45 nm and bfloGFPc1 = 493 ± 55 nm for absorbance, and bfloGFPa1 = 512 ± 62 nm and bfloGFPc1 = 521 ± 58 nm for fluorescence emission. The excitation spectrum of bfloGFPa1 peaked at 500 nm (FWHM = 38 nm) for emission fixed at 512 nm (Table 1), while the excitation spectrum for bfloGFPc1 was below detection limits of the spectrophotometer.

bfloGFPc1 and bfloGFPa1 crystallized with 8 and 2 molecules in the asymmetric unit, respectively. In both cases, the common oligomeric units were dimers. The crystals were brightly colored (greenish for bfloGFPa1 and yellow for bfloGFPc1), and retained their color throughout the duration of the data collection at 100 K. bfloGFPa1 crystals diffracted x-rays to 1.35 Å resolution while bfloGFPc1 crystals yielded measurable diffraction to 1.9 Å resolution. Phasing of bfloGFPc1 (8 molecules per asymmetric unit) via molecular replacement afforded high quality electron density maps for monomers A–D while the electron density for monomers E–G were of a lower quality. In the case of bfloGFPa1 (2 molecules per asymmetric unit), electron density maps for both monomers were of a high quality. Model building and refinement yielded final GFP structures exhibiting electron density for all amino acid residues except residues 1 and 182–187 (bfloGFPa1 monomer A), residues 1 and 219 (bfloGFPa1 monomer B), and residues 1–2 (bfloGFPc1a) (Table S1).

### Spectroscopic properties

Molar extinction coefficients measured for bfloGFPa1 and bfloGFPc1 were 120,900 M^−1^ cm^−1^ and 98,800 M^−1^ cm^−1^, respectively; both coefficients were significantly larger than the extinction coefficients of copGFP (70,000 M^−1^ cm^−1^)^37^ and that the one of eGFP (reported between 55,000–57,000 M^−1^ cm^−1^^5^ and indeed measured here at 56,000 M^−1^ cm^−1^) (Table 1). bfloGFPs' extinction coefficients were significantly greater these of commonly engineered FPs^9^,^29^,^38^ and similar in magnitude –but not as high- as to the one of the cnidarian *Renilla reniformis* (sea pansy) GFP with a value of 133,000 M^−1^ cm^-1^ 39,40,41.

The QE of eGFP calculated in our laboratory was 0.65, which was also comparable to the published value of 0.60^42^. In contrast, QEs were drastically different for *Amphioxus* GFPs compared to other GFPs; bfloGFPc1 has a very low QE of 0.0015, while bfloGFPa1 exhibited the maximum possible QE of 1.04 ± 0.05 (Table 1). These spectral properties remained unchanged in His-tagged bfloGFPs.

Due to a very low QE, bfloGFPc1 is quite dim with a measured brightness of 0.148 (mM * cm)^−1^. In contrast, bfloGFPa1 is exceedingly bright with a measured value of 121 (mM * cm)^−1^ (Table 1). To the best of our knowledge, this value makes bfloGFPa1 the brightest natural GFP characterized to date, well above commercial GFPs considered to be very bright, including the engineered EmGFP with brightness of 39 (mM * cm)^−1^ 42, and the recently reported native pmimGFPs from copepod with brightness of 70 (mM * cm)^−1^ 43, and the native VFP from coral with brightness of 107 (mM * cm)^−1^ 38.

### pH-dependence of GFP fluorescent properties

**Figure 1:**
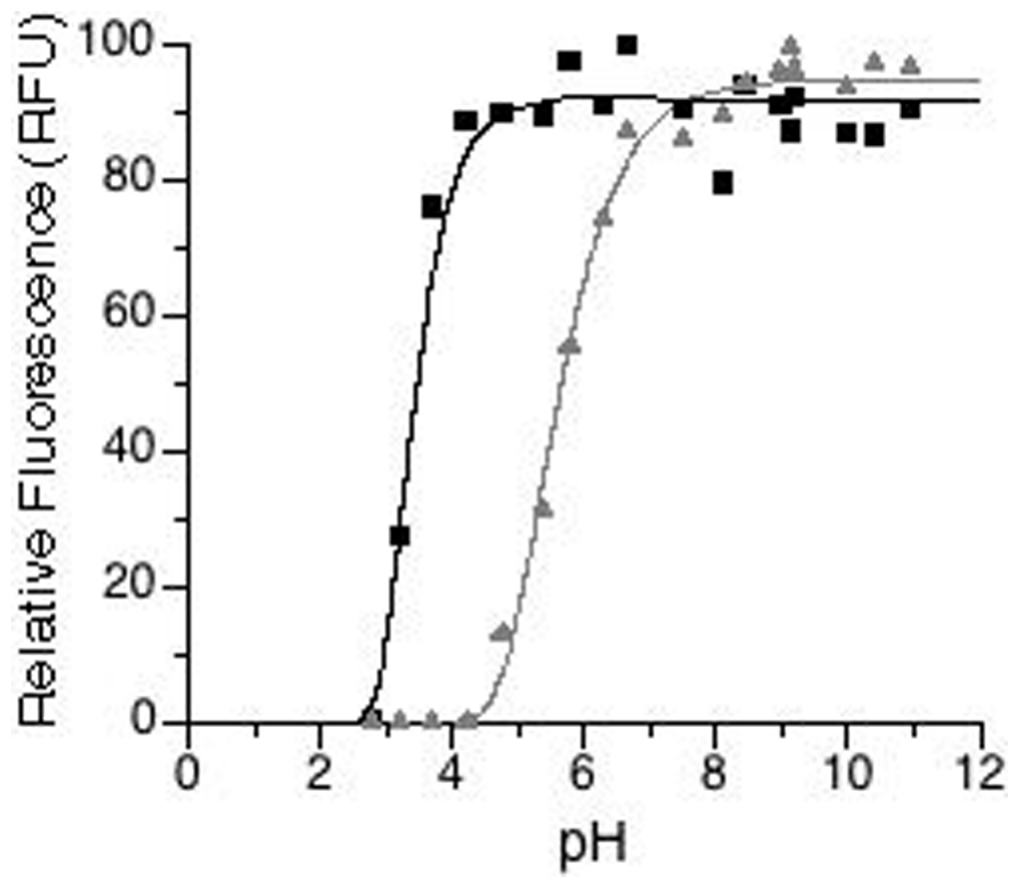
Change in relative fluorescence intensity (RFU) with pH for eGFP (grey triangles) and bfloGFPa1 (black squares). Raw data (symbols) are fitted with a double-exponential (sigmoidal) dose-response model from which the pKa was calculated.

The pK_a_ could not be determined for the bfloGFPc1 fluorescence due to the barely detectable fluorescence well below the detection limit for conventional spectroscopic instrumentation. As for bfloGFPa1, it exhibited a pK_a_ of 3.0 for its fluorescence, exhibiting high intensity for pH values ranging from 3.5 to 11 (Fig. 1). Notably, the high intensity for the acidic pH range is unusual compared to other GFPs such as eGFP. For instance, the pK_a_ for eGFP fluorescence calculated in our laboratory was 5.7, only slightly lower than the previously reported value of 6.0^42^ (Table 1, Fig. 1).

### Re-oxidation and refolding

Re-oxidation and refolding rates for bfloGFPa1 were measured and compared to those for eGFP (Table 2). In both fast and slow phases, the rate of reoxidation of the bfloGFPa1 chromophore occurred two times faster than that of the eGFP chromophore. In the case of refolding, the fast phase occurred at approximately the same rate for both bfloGFPa1 and eGFP, while the slower phase occurred three times faster for bfloGFPa1 compared to eGFP (Table 2).

### Three-dimensional architecture

Despite biochemical characteristics that were clearly different from one another (see above), we found that bfloGFPa1 and bfloGFPc1 shared essentially identical global tertiary and quaternary structures with a root mean square deviation (rmsd) for backbone atoms of 0.9 Å; for this reason, the results described in this section will use “bfloGFPs” to refer to both bfloGFPa1 and bfloGFPc1. bfloGFPs possess the classic 11-stranded β-barrel structure observed for all GFPs crystallized to date. In bfloGFPs, the barrel is capped by a combination of inter-strand loops at the bottom, and inter-strand loops and helices at the top. Following the central chromophore bearing helix (α1) there is a short helix at the top of the barrel (α2), common to all GFP structures, juxtaposed next to a third short helix (α3) that appears unique to *Amphioxus* and crustacean copepod GFP-structures (pdbid = 2G3O). The crustacean copepod GFP shares up to 35% amino acid sequence identity with bfloGFPs (compared to <19% between cnidarian GFPs and bfloGFPs), and is in fact also the closest GFP-containing evolutionary relative to *Amphioxus*^32^. GFPs from copepod and amphioxus bear high structural similarity; the rmsd of backbone atoms between bfloGFPa1 and copepod copGFP is 1.1 Å. The most obvious differences appear to be associated with the conformation of the loop and helical regions capping the top of the GFP β-barrel (Fig. S2).

### Chromophore environment

While globally the architectures of bfloGFPa1 and copGFP displayed a high degree of structural similarity, the local environments of the chromophores in both cases were highly divergent, with only 7 out of 19 chromophore-contacting residues strictly conserved (Fig. S3). Arg 195 and Glu 210, conserved across all GFPs described to date, were also present in bfloGFPa1, as were Tyr 104 and Arg 88, that latter of which form hydrogen bonds with the chromophore's peptidic and imidazolinone carbonyls, respectively. Phe 155, Phe 102, and Tyr 62, characteristic of copepod GFP, were also observed in amphioxus GFPs. These three aromatic residues contributed to the well-packed hydrophobic surface encapsulating the bfloGFPa1 chromophore. However, significant variation in chromophore contacting residues includes Cys 139 (GFPa1) to Thr 138 (copGFP), Val 197 (GFPa1) to Cys 197 (copGFP), Trp 157(GFPa1) to Arg 156 (copGFP), Ser 141 (GFPa1) to Glu 140 (copGFP), and Pro 55 (GFPa1) to His 54 (copGFP). Some of these residues appear critical for the overall energetic stability of the protein since substitution of one of them using site-specific mutagenesis lead to protein precipitation, especially upon exposure to blue excitation light (see Mutagenesis section in Discussion).

**Figure 2:**
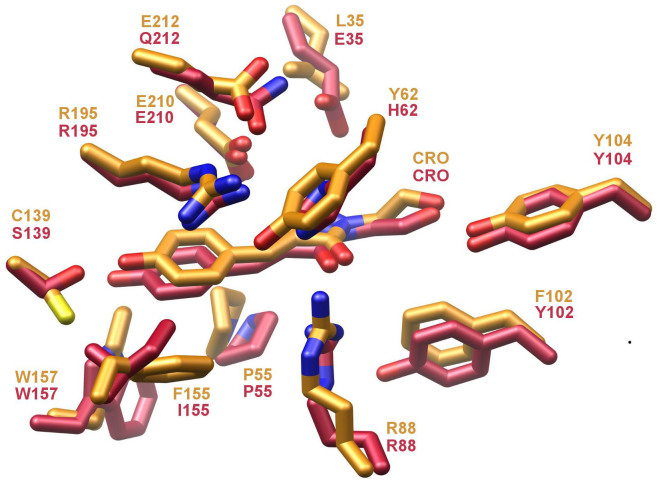
Overlay of bfloGFPa1 and bfloGFPc1 chromophore sites. Side chains and chromophores (CRO) are shown in ball-and-stick representation with carbon atoms colored gold (bfloGFPa1) and red (bfloGFPc1). bfloGFPc1 bears the Arg 195 and Glu 210 conserved across all GFPs as well as the Tyr 104 and Arg 88 involved in making H-bonds with the chromophore. As for the Phe 155, Phe 102, and Tyr 62, they were substituted in bfloGFPc1 by Ile, Tyr, and His, respectively. The three other residues common to bfloGFPa1 and bfloGFPc1 were therefore unique to amphioxus GFPs only, while found divergent in copGFP. These residues are Trp 157, Pro 55 and Leu 208, and could play a critical role in the unique biochemical characteristics/differences of the amphioxus GFPs as presented earlier (see also^32^). In particular, Trp 157 (Arg in copGFP) and Pro 55 (His in copGFP) form the base of the chromophore binding site where the phenolic ring of the chromophore sits, thus promoting Van der Waals contacts, especially with Pro 55.

When bfloGFPa1 is compared to bfloGFPc1, the degree of conservation between chromophore sites was 37% with only 7 out of 19 residues retained. However, only 4 out of 7 residues for each set were common to all three proteins (Fig. 2). Interestingly, we observed that Pro 55 (His in copGFP), conserved in both bfloGFPc1 and bfloGFPa1, was part of a cis peptide bond in bfloGFPa1 and a trans peptide bond in bfloGFPc1 suggestive that these two conformationally distinct states may play a critical role in modulating the pronounced differences in brightness between these two otherwise similar *Amphioxus* GFPs.

**Figure 3:**
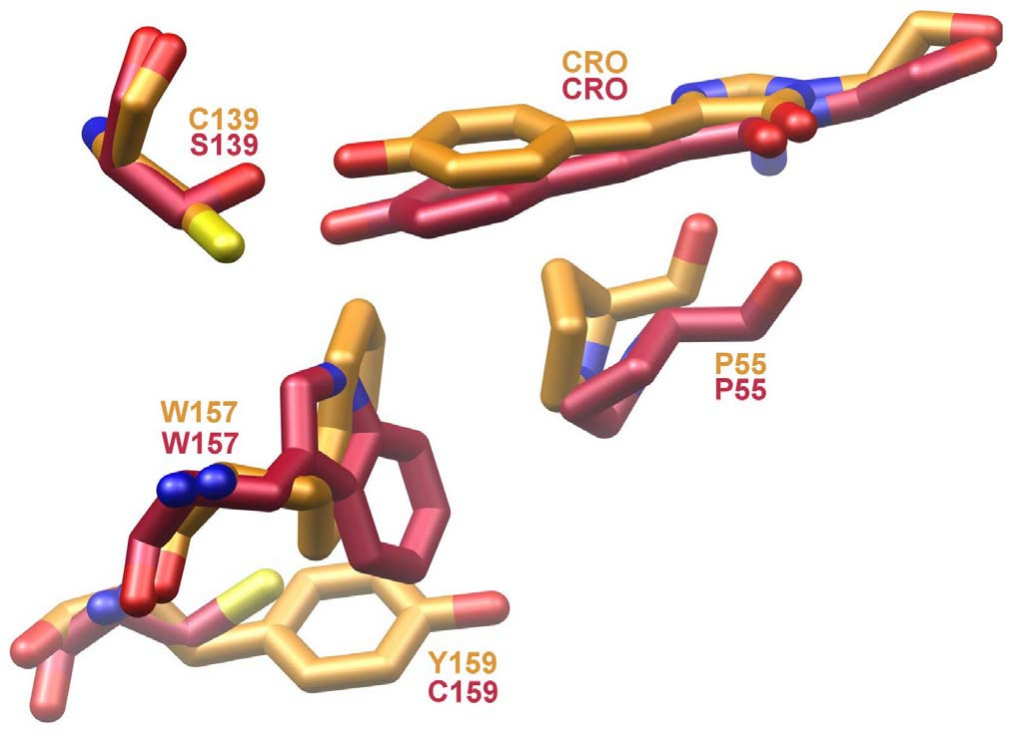
Ball-and-stick representation of key residues in bfloGFPa1 (gold) and bfloGFPc1 (red) that appear determinants of chromophore energetic stability.

Likewise, the indole ring of Trp 157, abutting the phenolic oxygen moiety of the chromophore, flips its relative orientation for bfloGFPa1 compared to bfloGFPc1. In bfloGFPc1, the Nε1 nitrogen of the Trp 157 forms a hydrogen bond with the phenolic oxygen while in bfloGFPa1 the ring flip negates a similar interaction (Fig. 3). These distinct rotamers for Trp 157 may correlate with what appears to be a well-stabilized edge-to-face interaction of the indole moiety with Tyr 159 in bfloGFPa1. In contrast, bfloGFPc1 bears a Cys at position 159 (Fig. 3). Finally, the chromophore of bfloGFPa1 is tilted 10° relative to the bfloGFPc1 chromophore. In total, the changes just described result in significant differences in chromophore cavity volumes between bfloGFPa1 and bfloGFPc1, the latter possessing a cavity 30% larger (265 Å^3^ vs. 386 Å^3^). Interestingly, the chromophore cavities of bfloGFPa1 and copGFP were similar in volume (265 Å^3^ vs. 280 Å^3^)^44^.

The x-ray crystal structure of the brightly fluorescent bfloGFPa1 exhibited two unique features that were different from the structures of the dimmer bfloGFPc1 and copGFP. The first property relates to the atomic displacement factors (ADP) or B-factors, which partially represent the thermal motion and disorder of a particular atom averaged across one or more crystals used for the dataset employed during coordinate and ADP refinement^45^. The average B-factor for the bfloGFPa1 chromophore atoms refined to a value of 9.2 Å^2^, which is much smaller than 39.2 Å^2^ for bfloGFPc1 or 26.8 Å^2^ for copGFP. While only partially indicative of thermal motion associated with any particular atom or group of atoms, the low B-factors for the atoms making up the chromophore in the bfloGFPa1 crystal suggests the chromophore possesses particularly low thermal motion with energy dissipation occurring primarily through fluorescence in bfloGFPa1.

**Figure 4:**
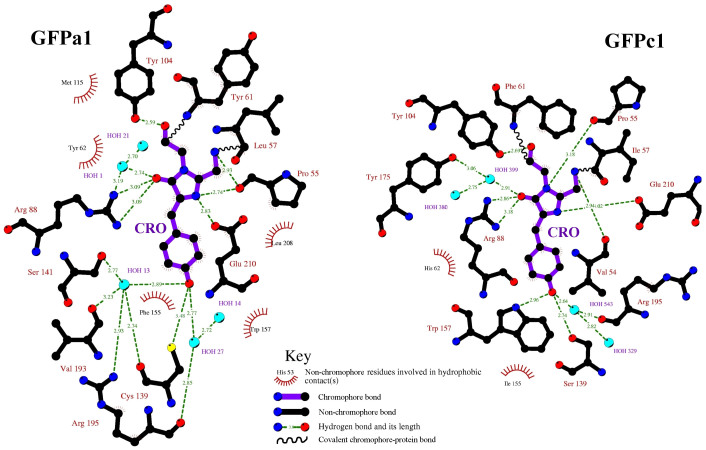
Ligplot diagram showing the difference in chromophore interacting residues between the brightly fluorescent bfloGFPa1 and the dimly fluorescent bfloGFPc1. Key hydrophobic interactions and covalent bonds are depicted and hydrogen bonding distances shown. The chromophore bonds are shown in purple while bonds in the surrounding residues are shown in black.

The second unique feature relates to the hydrogen-bonding network of the chromophores' phenolic hydroxyl groups. In the case of bfloGFPc1, copGFP, and many other GFPs, the phenolic hydroxyl moiety forms hydrogen bonds with one water molecule and two residues from the protein cavity, usually involving a Thr-Arg (copGFP), Ser-Trp (bfloGFPc1), or His-Thr (eGFP). In the case of brightly fluorescent bfloGFPa1, a Cys residue, Cys 139, resides proximal to and within hydrogen bonding distance of the chromophore's phenolic hydroxyl moiety. The remaining two hydrogen bonds form with water molecules anchored in the chromophore cavity (Fig. 4), which emphasizes the concerted role chromophore contacting residues play in modulating chromophore rigidity.

## Discussion

Despite more than a decade of GFP engineering directed at developing FPs with varied photonic properties, an understanding of the factors modulating “brightness” remain uncertain. Given the remarkably high degree of brightness associated with bfloGFPa1 and the unexpectedly low degree of brightness for its evolutionary cousin, bfloGFPc1, we investigated the atomic resolution architecture of both proteins with the aim of establishing a structural basis for this surprising difference in GFP brightness within a single organism, the cephalochordate *Amphioxus*.

In this study, we address the structural basis of brightness in GFPs by comparing two contrasting GFP structures from *Amphioxus* (bfloGFPs) with the structure of the evolutionarily related copepod GFP (copGFP). Two logical mechanisms for tuning brightness and QE emerged from this comparative analysis. Specifically, the mechanisms relate to the extreme conformational rigidity of the chromophore in the exceptionally bright *Amphioxus* GFP, bfloGFPa1, and its alteration of the hydrogen-bonding environment surrounding the chromophore's phenolic hydroxyl moiety.

QE positively correlates with increased rigidity of chromophores and in GFPs, this rigidity is provided by its protective β-barrel structure^13^. The emission of increasingly brighter fluorescence indeed seems to strongly correlate with the enhanced stiffness of the encapsulated chromophore, by preventing dissipation of the excited state energy through isomerization of neighboring residues during the excited state^14^,^46^,^47^. Enhanced stiffness of the chromophore, which is combined with a slight tilt in the bright bfloGFPa1, could also decrease the non-coplanarity of the residues, which is known to increase QE in GFP^11^,^48^,^49^. This is also supported from studies on the photochromic GFP Padron in which the photoswitched transition between fluorescent and non-fluorescent states is associated with the combination of both tilting and twisting of the two chromophoric rings relative to one another^50^. Such process takes places without affecting the planarity *per se*, indicating that high fluorescence QE is not necessarily associated with planar chromophoric rings^50^. This could explain the fact that the bfloGFPa1 has maximum QE while also have 6.8° coplanar difference between the chromophoric rings. The maximum QE in bfloGFPa1 seems to be related instead to a combination of other factors related to the slight 10° tilt of the chromophore when pushed into its pocket (no twisting was detected), and/or to the vibrational freedom of the rings, as shown by analysis of the B-factor (see section below). Comparison to other maximum QE is limited: the Verde fluorescent protein (VFP) from the coral *Cyphastrea microphthalma* is the only other native GFP reported so far with 100% QE^38^, although with lesser brightness than bfloGFPa1 because of lower extinction coefficient, being 107,000 M^−1^ cm^−1^^38^ compare to 120,900 M^−1^ cm^−1^ for bfloGFPa1. Crystallography data from VFP are currently not available to address similarities or differences with structural features of bfloGFPa1.

The crystallographic atomic displacement factor (or B-factor) is a partial measure of this rigidity, using as a proxy the level of flexibility induced by thermal energy. The B-factor is therefore resolution dependent, and when comparing structures resolved at the same crystallographic resolution, the lower the B-factors of a particular groups of atoms, the more rigid the associated structure. We screened the PDB for GFP structures refined to a similar resolution as bfloGFPa1 and found 15 of them (Table S2) also studied elsewhere^51^. We then analyzed the average temperature factor of the atoms comprising the chromophores of these GFPs and found that the B-factor ranges between 9.5 Å^2^ (2CD1) and 21.1 Å^2^ (2DUG) (Table S2). All these GFPs are reported with QE similar to the original GFP template and thus well lower than 100%, thus reinforcing the observation that the lower the B-factor, the greater the stiffness, and the greater the QE. Indeed, in comparison, the 100% QE bfloGFPa1 bears a relatively low average B-factor of 8.7 Å^2^ for the atoms making up its chromophore, suggesting that the chromophore is very rigidly restrained in the chromophore pocket in bfloGFPa1. Such trend was in fact observed specifically at, or around, the chromophore per se since analyses of the B-factor around residue 60 were about 40 Å^2^ for bfloGFPc1 (viz. clearly indicative of a high flexibility region), while being <10 Å^2^ for bfloGFPa1. The low B-factor for bfloGFPa1 clearly indicates that the chromophore and its surrounding have limited structural flexibility (hence high stiffness), which is known widely in the literature to be associated with greater QE (e.g.,^29^,^46^,^52^).

If the B-factor clearly plays an important role driving the QE in GFPs, it is also likely not the only one, and several factors might have to act in synergy for reaching maximal QE. This might be indicated for example by the Glycine-containing mutant 3CB9, which corresponds to a redox sensitive GFP with insertion of an extra amino acid, in this case arginine^51^. In this mutant, the QE is reported low despite however particularly low B-factor of 6.2 Å^2^, which is indicative of high stiffness of the chromophore and thus high QE. This suggests that there is another factor that affects QE, which could be the chromophore pocket. Indeed, we believe that the conformational rigidity of the bfloGFPa1 chromophore may be in part the result of the smaller volume of its chromophore pocket relative to its very dim cousin bfloGFPc1. Nevertheless, the volume of the bfloGFPa1 chromophore pocket is approximately 65% smaller than the same pocket calculated for the static x-ray crystal structure of eGFP, and roughly equivalent to the one found in an evolutionarily related GFP, namely copGFP, yet both of these latter GFPs are 5–10 times dimmer than bfloGFPa1. This observation indicates that volume of the chromophore pocket alone cannot be the only factor modulating QE and ultimately brightness. A restricted pocket size would likely contribute to maintaining the chromophore in a strained, high-energy conformation, thus contributing to an increased efficiency for photon transfer and fluorescence, while also increasing protection of the chromophore from quenching agents^53^,^54^.

Through comparisons of GFP structures possessing widely varying spectral properties but isolated from the same organism, we were able to clearly define other structural features not obvious from sequence alignments alone that likely contribute to the modulation of QE and brightness in these naturally evolved FPs. In the *Amphioxus* GFP structures elucidated and described here, it is clear that the chromophore packs against a conserved Pro, Pro 55, that assumes distinct backbone conformational states in each GFP; in the intensely bright GFP, bfloGFPa1, the peptide bond, Ile 54 – Pro 55, adopts a cis orientation while the same peptide, Ile 54 – Pro 55 adopts a trans orientation in the very dim bfloGFPc1. These divergent backbone conformations shift the positions of the encapsulated chromophore helical segment, resulting in the formation of a single hydrogen bond between the imidazolinone nitrogen and the backbone carbonyl oxygen of the Pro 55 in bfloGFPc1. Such an alteration of the hydrogen bonding patterns of the β-barrel with the chromophore would expectedly lead to change(s) in the chromophores' emission efficiencies and the brightness of the resultant fluorescence^12^.

An additional set of changes in the chromophore environments when comparing bfloGFPa1 and bfloGFPc1 centers around the rotameric state of the chromophore contacting Trp 157. In bfloGFPa1, this rotation disrupts a hydrogen bond between the indole NH of Trp 157 and the hydroxyl moiety of Tyr 159 that occurs in bfloGFPc1. This structural distortion results in the phenyl portion of Trp 157 abutting the phenolic end of the chromophore, thus increasing the hydrophobicity of the bfloGFPa1 chromophore environment compared to that of bfloGFPc1. This rotation appears to be stabilized by the edge-to-face interaction between the aromatic moieties of Trp 157 and Tyr 159. Without an actual experimentally determined three-dimensional structure, this type of second tier interaction can be challenging to identify unequivocally.

Currently, genetic engineering of GFPs is focusing more and more on improving the QEs but remains an ongoing challenge compared to modifying the biochemical and/or biophotonic properties of FPs^3^,^13^,^55^. Therefore, bfloGFPa1 may represent an alternative evolutionarily optimized GFP with which to pursue the directed evolution of other desirable parameters, including oligomeric state, greater tolerance to acidic pH, better resistance to bleaching, faster folding and chromophore maturation, large extinction coefficient, and broad excitation and emission spectra. Given its perfect quantum efficiency, its broad fluorescence pKa range, and its relatively fast folding and chromophore maturation rates, *Amphioxus* bfloGFPa1 appears to be an ideal candidate for future applications and engineering efforts. This has been materialized already by the use of bfloGFPa1 in bioassays where increased brightness and biochemical stability were key in providing signal not otherwise available using conventional FPs^56^,^57^. In addition, a GFP from *Branchiostoma lanceolatum*, the amphioxus species from Europe, is now commercially available (under the name of lanGFP) and showing about 4x-increased brightness compared to eGFP^58^, thus comparable to bfloGFPa1. This strongly suggests that amphioxus GFPs indeed hold attractive promises for future use in an extended range of applications than currently available.

We used conventional techniques of mutagenesis of critical residues in a first attempt to demonstrate the role of key residues from the chromophore pocket that are associated with high versus low brightness or quantum efficiency. We performed site directed amino acid substitutions on bfloGFPa1 based on differences with bfloGFPc1 and copGFP. bfloGFP mutants were generated using the QuikChange (Stratagene, San Diego, CA) PCR-based method. Mutant enzymes were expressed and purified as described in this study for wild-type bfloGFP. In addition to wtGFP controls, we completed five different single mutations chosen to provide further understanding of the relationship amongst the diverse GFPs within amphioxus^34^. We then attempted to perform spectroscopic and biochemical comparative analysis on the five mutants using the exact same proteomic and spectroscopic protocols as used for bfloGFPa1. The mutants were P55H and C139S (P55 and C139 are both unique to GFPa1), F155L and Y62H (F155 and Y62 are common to GFPa1 and copGFP and contribute to chromophore encapsulation), and R195A (common to all GFPs and responsible for hydrogen bonding in the chromophore cavity). These mutations thus involve residues with significant role in providing energetic stability to the chromophore (Fig. 4).

The five bfloGFPs mutants were successfully expressed and exhibiting strong fluorescence, except for C139S, F155L and R195A that showed relatively lower fluorescence intensity, yet always with a spectrum similar to that of bfloGFPa1. The absorbance spectrum was also measurable for each mutant, and remained similar amongst all bfloGFPs, yet showing relatively greater values for F155L. Complete and detailed biochemical and spectroscopic characterization of the mutants could however not be performed because all mutants were unstable, constantly precipitating out of solution, which was otherwise not observed for the wtGFP control, or when maintained in darkness. Consequently, determining accurate protein concentration necessary for calculation of the extinction coefficient, for performing biochemical experiments, and for interpretation of the spectroscopic measurements from the bfloGFPa1 mutants was not possible using the current protocols. However, these data suggests that the five mutations we performed in the chromophore pocket do not seem to qualitatively affect the spectral characteristics of bfloGFPs, while clearly indicating that each of the substituted residues (proposed to each have a critical function in the chromophore pocket) plays a key role in contributing to energetic stability of the whole protein.

Amphioxus contains 16 bfloGFPs organized in six clades^34^, each showing various substitutions of one (or more) of the residues that we experimentally mutated in this study. The co-occurrence of many different GFPs in one single organism therefore indicates that the unbalance in energy due to substitution of the residues considered here must be compensated in the natural system by additional substitutions in other areas of the protein, in order to provide protein stability. This is clearly a possible scenario considering that protein sequence identity amongst clades varies from 49 to 65%^34^, and that FPs in general appear to have distinct regions (the relatively conserved central chromophore region versus the N- and C- terminal variable regions) with divergent evolutions and different molecular functions^59^. At this stage, genetic engineering of bfloGFPa1 appears attractive, yet requiring the screening of tandem substitutions and/or alternative protocols in order to preserve stability of the protein in solution^12^,^13^. Performing mutations of residues other than the five ones presented here would be key in future engineering studies, especially considering that mutations leading to other colors of fluorescence are different from the ones we tested^9^, thus giving the prospect to preserve maximal quantum efficiency for engineered bfloGFPa1 with different emission spectra.

## Methods

### Cloning, protein expression, and purification

*Amphioxus* bfloGFPa1 and bfloGFPc1 were cloned and purified as previously reported^34^. eGFP and YFP were kindly provided by Air Force Research Laboratories (WPAFB, Ohio), sub-cloned into pET24b, and purified as described for *Amphioxus* GFPs^34^. Spectral characteristics (absorbance and fluorescence) of all these fluorescent proteins were measured using a spectrophotometer SpectraMax M2 (Molecular Devices, Sunnyvale, CA) with complete scanning of the spectral range (comprise between 400–800 nm) but expressed here as peak and full width at half maximum (FWHM) of each spectrum.

### Protein concentration and extinction coefficient calculation

Protein concentrations were calculated using the extinction coefficient of the chromophore after denaturation in 0.1 N NaOH (44,000 M^−1^ cm^−1^ at 446 nm)^39^. Absorbances of bfloGFPa1, bfloGFPc1 and eGFP were measured with the spectrophotometer and extinction coefficients calculated according to the Beer Lambert law, A = e*l*c, were “A” is absorption of a given wavelength of light, “e” is the molar extinction coefficient of a certain species, “l” is the path-length, and “c” is the concentration of that given sample.

### Quantum efficiency calculation

Fluorescence quantum efficiency (QE) of bfloGFPa1 and bfloGFPc1 was measured from six independent replicate expression batches, with and without His-tag, using the method of relative actinometry. Emission of the GFPs was then expressed relative to a known standard with the same absorption at the excitation wavelength of 450 nm, keeping all instrumental conditions identical^60^. Here, fluorescein in 0.1 N NaOH was used as a standard since its QY is 0.90 at 25°C^60^, while eGFP was used to inter-calibrate our experimental setup and measurements in comparison to published values for eGFP. All samples were maintained in a dilute state adjusted to an absorbance of 0.11 at 450 nm for accurate comparisons. The area under the emission curve extending from 460 to 800 nm was then integrated and the following formula used for calculating the quantum yield: 
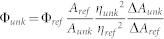
where “F” is the fluorescence quantum yield, “A” is the absorbance of the unknown and standard at the exciting wavelength, “h” is the refractive index of the solvent, and “DA” is the area of the 460–800 nm emission. The refractive index values were h = 1.3576 for the 0.1 N NaOH standard, and h = 1.5442 for the 50 mM Tris-HCl (pH 8), 400 mM NaCl sample buffer, which is the value for a solution in which NaCl is the predominant analyte^61^.

### GFP refolding and reoxidation

Refolding and re-oxidation experiments were performed as previously described^62^. Briefly, pure GFP in 50 mM Tris-HCl, pH 8, 400 mM NaCl, 1 mM DTT was diluted to 2 mg/ml in 8 M Urea, 1 mM DTT for refolding experiments, and 8 M Urea, 1 mM DTT, 5 mM Dithionite for re-oxidation experiments. The samples were heated to 95°C for 5 min, cooled to room temperature (~23°C), and diluted 1:100 in renaturation buffer (35 mM KCl, 2 mM MgCl_2_, 50 mM Tris-HCl, pH 8, and 1 mM DTT). The SpectraMax M2 plate reader equipped with a 495 nm excitation filter was used to follow refolding and re-oxidation, using as a proxy the fluorescence intensity of the GFPs. Samples were excited at 475 nm and fluorescence emission recorded at 508 nm (eGFP) or 516 nm (bfloGFPa1) every second for 3,000 sec (50 min). Both refolding and re-oxidation curves were modeled employing a two-phase exponential equation using Prizm 4.00 (GraphPad). The equation was as follow: Y = P + C_F_*exp(−K_F_*X) + C_S_*exp(−K_S_*X), where “P” is the Plateau value reached at infinite time for “X”, “C_F_” and “C_S_” are the two time constants for the Fast and Slow half-life, respectively, and “K_F_” and “K_S_” are the rate constants for the Fast and Slow half-life, respectively. The Fast and Slow half-lives were then computed as ln(2)/K_F_ and Ln(2)/K_S_, respectively. Parameters of the equations were estimated by iteration from the raw values of fluorescence, following the nonlinear estimation method of Quasi-Newton, while the resulting best fit model is indicated by the R corrected for non-linear systems^63^.

### pH titration

*Amphioxus* GFP fluorescence at various pH values was evaluated using citric acid - sodium citrate (pH 3–5), sodium phosphate (pH 5–6), Tris-HCl - Tris-Base (pH 6–9), and glycine - NaOH (pH 9–12). Each sample consisted of concentrated GFP in a weakly buffered solution (~5 mM) diluted approximately 200-fold (to 50 nM final concentration) into 50 mM buffer at a given pH. The SpectraMax M2 plate reader with a 495 nm cut-off filter was used to record fluorescence emission at 508 (eGFP) and 516 (bfloGFPa1) upon excitation at 475 nm. Absorbance spectra of each sample were subsequently recorded and used as internal controls for calibration of protein concentrations.

### Crystallization

bfloGFPc1 crystals were grown overnight by vapor diffusion at 4°C in 2.0 μL drops, consisting of 1 μL crystallization reservoir [28% (w/v) polyethylene glycol 8,000, 1 M NaCl, 100 mM HEPES-Na+ (pH 7.5)], and 1 μL protein solution [419 μM bfloGFPc1]. Diffraction data were collected at the Berkeley Advanced Light Source (ALS) synchrotron beamline 8.2.2 on a Quantum Q315 CCD detector. bfloGFPc1 crystallized in spacegroup C2, a = 158.76 Å, b = 130.46 Å, c = 106.33 Å, a = g = 90°, b = 128.39° with eight monomers in the asymmetric unit. Data were indexed, integrated, and scaled to 1.95 Å with HKL2000^64^. bfloGFPa1 crystals were grown overnight by vapor diffusion at 4°C in 2.0 μL drops, consisting of 1 μL crystallization reservoir [30% (w/v) polyethylene glycol 4,000, 3% (v/v) isopropanol, 100 mM citric acid – sodium citrate (pH 5.5)], and 1 μL protein solution [336 μM bfloGFPa1]. Diffraction data were collected at the ALS synchrotron beamline 8.2.1 on a Quantum Q315 CCD detector. GFPa1 crystallized in spacegroup C222(1), a = 59.25 Å, b = 125.57 Å, c = 106.46 Å, a = b = g = 90° with two monomers in the asymmetric unit. Data were indexed, integrated, and scaled to 1.35 Å with HKL2000^64^. Structure elucidation process is described in supplementary information.

### Structure elucidation

Phase determination of bfloGFPc1 was accomplished via molecular replacement (MR) using the program Phaser^65^. The MR search model was a mixed model^66^ based on the structure of copepod GFP (2G3O). MR phases were used for manual model building of the bfloGFPc1 tertiary structure in Coot^67^. Iterative stages of building and refinement were carried out using Coot and CNS^68^, respectively. Refinement was completed imposing restrained non-crystallographic symmetry between the 8 GFP monomers. The final structure was evaluated with PROCHECK^69^. The bfloGFPc1 structure had 92.6% and 7.4% of residues in the most favored and allowed regions of the Ramachandran plot, respectively. The final structural coordinates and structure factors were deposited to the Protein Data Bank under PDB ID 3GJV.

Phase determination of bfloGFPa1 was accomplished via molecular replacement (MR) using the program Phaser. The MR search model was the refined structure of bfloGFPc1. MR phases were used for manual model building of the bfloGFPa1 tertiary structure in Coot^67^. Iterative stages of building and refinement were carried out using Coot and CNS^68^, respectively. The final structure was evaluated with PROCHECK^69^. The bfloGFPa1 structure had 91.5% and 8.5% of residues in the most favored and allowed regions of the Ramachandran plot, respectively. The final structural coordinates and structure factors were deposited to the Protein Data Bank under PDB ID 3GIH.

## Author Contributions

D.D.D. designed research; E.K.B. and J.E.H. performed research; J.P.N. provided support for crystallization and x-ray data collection; E.K.B. and D.D.D. wrote the paper; all authors read and edited the manuscript. The authors declare no conflict of interest.

## Additional information

**Crystal structures deposition** Structures of bfloGFPa1 and bfloGFPc1 were deposited at the RCSB Protein data bank under “Crystal structure of amphioxus green fluorescent protein, GFPa1, andGFPc1”, with assigned RCSB IDcode rcsb061402 and rcsb061411, respectively.

## Supplementary Material

Supplementary Information Suppl Tables and Figures

## Figures and Tables

**Table 1:** Photonic properties and pKa values for eGFP, bfloGFPa1 and bfloGFPc1

	eGFP	bfloGFPa1	bfloGFPc1
Absorbance Maximum Peak (nm)	488	497	493
FWHM (nm)		45	55
Fluorescence Maximum Excitation Peak (nm)	488	500	n.d.
FWHM (nm)	35–45	38	
Fluorescence Maximum Emission Peak (nm)	508	512	521
FWHM (nm)	30–40	62	58
Extinction Coefficient (M^−1^ cm^−1^) (per chain)	56,600^70^	120,900	98,800
Quantum Yield (%)	60^5^	104 ± 5	0.15 ± 0.1
Brightness	34	120.9	0.148
pKa	5.65/5.9^5^	3.0	n.d.

n.d.: not determined.

**Table 2:** Kinetics parameters associated with refolding and reoxidation of eGFP and bfloGFPa1 when modeled with a two-phase exponential equation

	Refolding		Reoxidation	
	eGFP	bfloGFPa1 bfloGFPa1	eGFP	bfloGFPa1
Emission (nm)	508	516	508	516
K1 (s^−1^)	5.02 × 10^−4^	6.83 × 10^−4^	3.83 × 10^−4^	6.67 × 10^−4^
K1_std. error_	7.20 × 10^−6^	4.08 × 10^−6^	1.20 × 10^−5^	7.33 × 10^−6^
K2 (s^−1^)	7.63 × 10^−3^	2.18 × 10^−2^	5.07 × 10^−3^	1.16 × 10^−2^
K2 _std. error_	2.01 × 10^−4^	2.69 × 10^−3^	7.15 × 10^−4^	1.72 × 10^−3^
T1_(1/2)_ (sec)	1,380	1,014	1,812	1,040
T2_(1/2)_ (sec)	90.89	31.82	136.70	59.93
r^2^	0.9990	0.9992	0.9981	0.9984
