# Supplementary material for: Spectral and structural comparison between bright and dim green fluorescent proteins in Amphioxus
Source: Sci Rep. 2014 Jun 27;4:5469. doi: 10.1038/srep05469 (PMC4073121; doi:10.1038/srep05469)

SUPPORTING INFORMATION ONLINE
Scientific Reports

Spectral and structural comparison between bright and dim green fluorescent proteins in Amphioxus

Erin K Bomati, Joy E Haley, Joseph P Noel and Dimitri D Deheyn

Supplemetary tables

Table S1. Crystallographic statistics associated with characterization of bfloGFPa1 and bfloGFPc1 structures.

	Statistic	Sub Category	bfloGFPa1	bfloGFPc1
DATA COLLECTION	Space group		C 2 2 2(1)	C 2
	Cell Dimensions	a, b, c,	59.25, 125.58, 106.47	158.76, 130.46, 106.33
		α, β, γ (°)	90.0, 90.0, 90.0	90.0, 128.4, 90.0
	Resolution (Å)*		50.0 - 1.35(1.4 - 1.35)	50.0 - 1.95 (2.02 – 1.95)
	R_{sym}		4.7 (29.1)	5.5 (51.3)
	I/σ		37.9 (3.3)	34.3 (2.7)
	Completeness (%)		97.4 (83.9)	99.7 (98.2)
	Redundancy		6.7 (5.2)	5.3 (4.9)
REFINEMENT	Resolution (Å)		1.35	1.95
	No. Reflections		87211	123290
	R_{work}/R_{free}		.2939/.3214	.2944/.3216
	No. of atoms	Protein	3357	13224
		Ligand	38	152
		Water	501	581
	B-factor	Protein	13.2	41.7
		Ligand	9.0	42.0
		Water	25.2	38.4
	R.m.s. deviations	Bond angles (°)		
		Bond lengths (Å)		

* Number in parentheses refers to highest shell

Table S2: List of GFPs found with similar crystallographic resolution as bfloGFPa1, and their average B-factor values from the atoms comprising the chromophore (from PDB database and Lohman and Remington 2008).

**PDB ID**	**B-factor**	**QE**
2DUG	21.1	<100%
2C9J	21.0	<100%
1XMZ	17.3	<100%
2QT2	15.9	<100%
2DUI	14.2	<100%
2DUE	13.6	<100%
2ZO6	12.6	<100%
1Q4A	11.7	<100%
1Q4E	11.0	<100%
2B3P	10.3	<100%
2GW3	10.2	<100%
2HRS	10.0	<100%
2O24	9.7	<100%
3CD1	9.5	<100%
3CB9	6.2	<100%
GFPa1	8.7	=100%

Supplementary Figures

Figure S1. Ribbon representation of the bfloGFPa1 dimer. Monomer A is colored blue, monomer B is colored rose. The GYG-derived chromophore is shown in space filling model with the carbon atoms colored black.

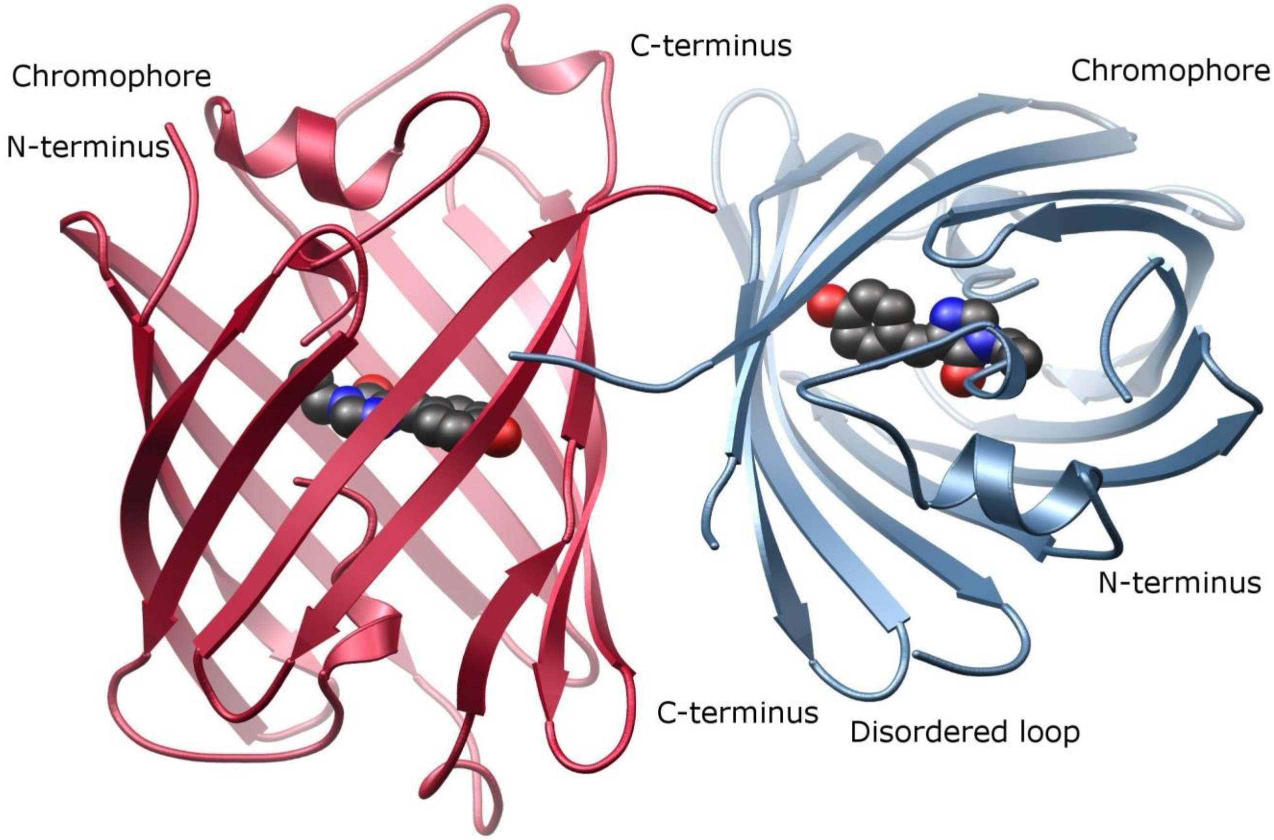

Figure S2. Overlay of structurally similar GFPs including bfloGFPa1 in gold, bfloGFPc1 in red, and copGFP (PDBid 2G3O) in blue. Chromophores are shown in ball-and-stick representation with carbon atoms color coordinated to match ribbon structure.

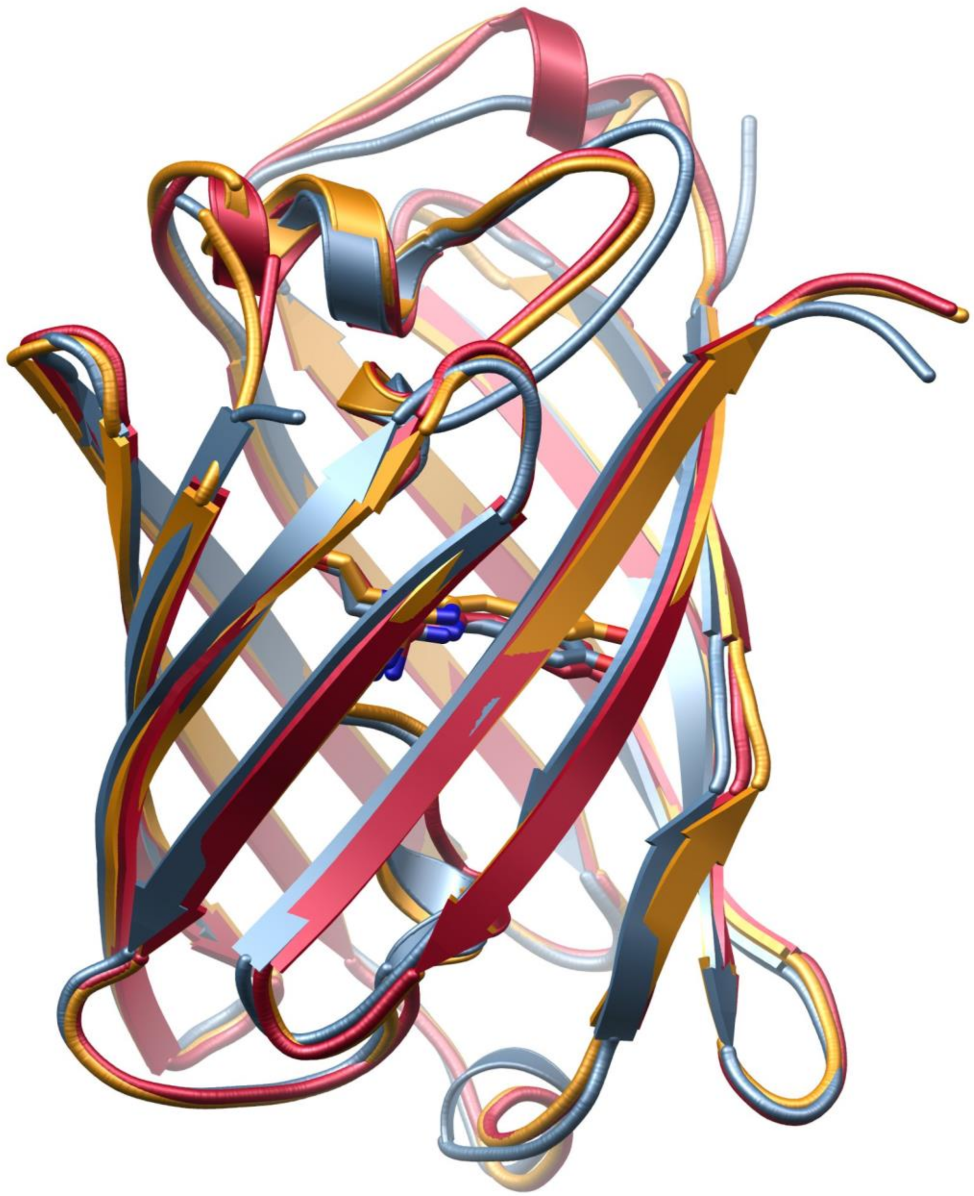

Figure S3. Overlay of bfloGFPa1 and copGFP chromophore sites. Side chains and chromophores (CRO) are shown in ball-and-stick representation with carbon atoms colored gold (bfloGFPa1) and blue (copGFP).

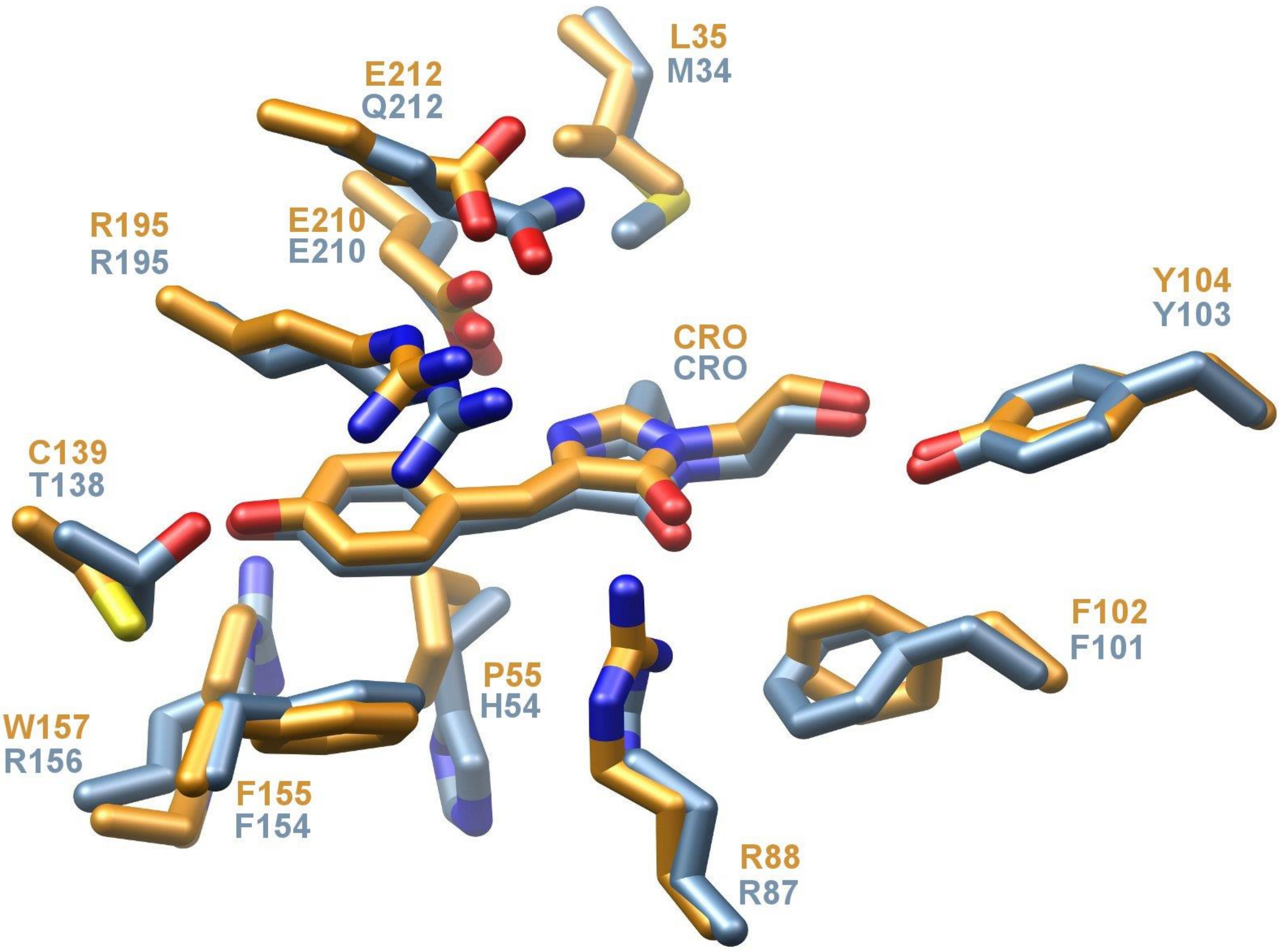

Supplement: Supplementary Information — Suppl Tables and Figures [file srep05469-s1.pdf]
